# Improvement of the rapid response system at an acute rehabilitation hospital in New Mexico

**DOI:** 10.2144/fsoa-2023-0162

**Published:** 2024-05-24

**Authors:** Reza Ehsanian, Jimmy To, David Mork, Melissa Owens, William F Gensler, Rebecca Dutton, John Henry Sloan

**Affiliations:** 1 Division of Pain Medicine, Department of Anesthesiology & Critical Care Medicine, University of New Mexico School of Medicine, Albuquerque, NM, USA; 2 Department of Rehabilitation Medicine, University of Minnesota School of Medicine, Minneapolis, MN, USA; 3 Lovelace UNM Rehabilitation Hospital, Albuquerque, NM, USA; 4 Division of Physical Medicine & Rehabilitation, Department of Orthopaedics & Rehabilitation, University of New Mexico School of Medicine, Albuquerque, NM, USA; 5 Manzano Medical Group, Albuquerque, NM, USA

**Keywords:** quality improvement, questionnaire, rapid response, rehabilitation hospital

## Abstract

**Aim:** Enhance the Rapid Response System (RRS) in a free-standing acute rehabilitation hospital (ARH) by improving announcements, crash cart standardization and role assignments. **Materials & methods:** Pre-intervention (PreIQ) and post-intervention questionnaires (PostIQ), conducted in English and utilizing a Likert scale, were distributed in-person to clinical staff, yielding a 100% response rate. The questionnaire underwent no prior testing. The PreIQ were disseminated in February 2021, and PostIQ in December 2022. **Results:** PostIQ illustrated the improvement of audibility and improved the clarity of roles. The training positively impacted the RRS in the ARH. **Conclusion:** This study highlights the value of continuous RRS improvement in ARHs. Interventions led to notable enhancements, emphasizing the need for sustained efforts and future research on broader implementation.

## Abstract (plain language summary)

Thousands of in-hospital and out-of-hospital cardiac arrests occur annually.

The Institute for Healthcare Improvement (IHI) launched the 100,000 Lives Campaign in 2004 which brought rise to the creation of rapid response teams (RRTs) within rapid response systems (RRS) to address rapid response cardiac arrest management.

Studies have determined that a successful RRS consists of an afferent limb with a proper triggering system, an efferent limb with an experienced multidisciplinary team, and a hospital culture that embraces the RRS.

In this study, the aim was to improve the RRS to rapid response events at a rehabilitation hospital in Albuquerque, New Mexico through quality improvement interventions in the following topics: rapid response announcements, crash cart stocks, and code response role assignments.

Questionnaires were given to hospital clinical staff before implementation of the study to assess their opinion of the RRS as well as after to assess the impact of the interventions.

Post-intervention questionnaire results illustrated improvements with rapid response announcements, code role assignments and hospital clinical staff training participation.

The limitations of the study included the small sample size and limited duration of follow-up after the intervention.

Continuous quality improvement can be an effective means of improving the RRS in hospitals, including rehabilitation hospitals.

Roughly 292,000 adult and 15,200 pediatric in-hospital cardiac arrests occur annually in the USA alone [1]. An alert system called ‘Code Blue’ has been established in hospitals that often signifies that a patient is undergoing cardiac arrest, thus requiring resuscitation and immediate medical attention [2]. The Institute for Healthcare Improvement (IHI) launched the ‘100,000 Lives’ campaign in December 2004 with the goal of saving 100,000 lives in US hospitals by 14 June 2006 [3]. One of the implementations to meet this goal was the creation of rapid response teams (RRT) within rapid response systems (RRS). The purpose of implementing RRTs was to address the ineffectiveness of the available resources at the time in rescuing Code Blue patients from spiraling into rapid deterioration [4].

Some studies have shown the benefits of having RRTs in addressing in-hospital cardiac arrests. Al-Qahtani *et al.* illustrated that the incorporation of RRTs was effective in reducing total hospital mortality while also improving the health outcomes of individuals who were discharged from the ICU [5]. In a separate study, it was found that activation of RRTs by more than 15 min after a patient became unstable independently correlated with increased risk of mortality [6]. Not all studies demonstrated positive outcomes, however. Hillman *et al.* discovered that while implementation of an RRS did increase emergency team calling, there was no major impact on unplanned ICU admissions, incidence of cardiac arrest or unexpected deaths [7]. This suggests that in addition to activating RRS’, uncovering ways to improve the effectiveness and efficiency of the system should also be considered.

Song *et al.* stated that successful RRS' requires an afferent limb with a proper triggering system, an efferent limb consisting of an experienced multidisciplinary team, and a hospital culture that embraces the RRS [8]. The afferent limb ‘triggers’ the RRTs when a patient's vital signs and laboratory values are abnormal, typically detected by a single or multi-parameter early warning system (EWS) [9]. Studies have shown that abnormal vital signs are typically prevalent 1–4 h before an in-hospital cardiac arrest occurs, suggesting the need for treating these abnormalities before irreversible mortality occurs [10]. The efferent limb involves RRTs that consist of critical care nurses, respiratory therapists and physicians who address the triggering system and perform an initial patient assessment. Experienced clinical staff leading RRTs have been shown to be crucial in obtaining positive patient outcomes [11,12].

Aside from having a strong afferent and efferent limb, a hospital culture that embraces the RRS has also been shown to be essential. A study by Loisa *et al.* analyzed the attitudes and barriers of RRT nurses to the RRS. They found that while RRT nurses found their work important, factors such as infrequent RRT participation, feeling overworked, and conflicts between the nurses and physicians were seen as barriers for a successful RRS [13]. Similarly, another study surveyed physicians and nurses on wards to identify the barriers associated with activation of RRS' and found that lack of familiarity, agreement with, and perceived benefit of the RRS is correlated with low self-reported adherence rates [14]. There have been studies that have investigated what makes a RRS successful. A common theme to having a successful system involves clear leadership and continuous quality improvement (QI) that emphasizes clinical competency of the RRT [^15-17^].

For this study, we applied these lessons of a successful RRS to our acute rehabilitation hospital (ARH). RRS evaluation for our hospital was done with hospital clinical staff and administration to identify barriers to a successful RRS. Many clinical staff indicated that they were unable to hear the RRS announcement. Random assessment of crash carts with the nursing manager also showed that crash cart equipment was not standardized. Discussion with clinical staff reported that roles were not clear during a rapid response as well. We created a QI study to address these shortcomings.

## Global aim statement

Improve the RRS to rapid response events at Lovelace UNM Rehabilitation Hospital by working with hospital and residency leadership through QI intervention and assessing the effectiveness these of interventions through questionnaires.

## Improvement aims

a) Improving announcements to help clinical staff hear rapid response activation throughout the ARH, b) improving crash carts by ensuring all crash carts are uniformly stocked, and c) improving role assignments to prevent confusion of clinical staff responsibilities during a rapid response.

## Materials & methods

### Study information

This was a QI study that was approved to be implemented at Lovelace UNM Rehabilitation Hospital in Albuquerque, New Mexico by the University of New Mexico Division of Physical Medicine and Rehabilitation under the guidance of hospital leadership (e.g. Medical Director, Chief Nursing Officer and Nursing Manager) and residency program leadership (e.g. Residency director, Residency Coordinator). The Human Research Review Committee at the University of New Mexico Health Sciences Center determined that this activity did not constitute human-subjects research (23–147).

### Facility

The free-standing acute inpatient rehabilitation facility for this study is in the state of New Mexico and capable of providing complex acute inpatient rehabilitation services. The rehabilitation hospital is the only hospital in New Mexico accredited by the Commission on Accreditation of Rehabilitation Facilities in six programs: Comprehensive Integrated Inpatient Rehabilitation Program, Inpatient Brain Injury Program, Inpatient and Outpatient Spinal Cord System of Care, Inpatient Stroke Specialty Program, Outpatient Medical Rehabilitation Program and Outpatient Medical Rehabilitation Program (children and adolescents). There are 62 licensed acute inpatient rehabilitation beds. Over 1000 inpatient admissions occurred in 2023. The hospital provides a full continuum of inpatient and outpatient rehabilitation care, including physical therapy, occupational therapy, speech and language pathology, rehabilitation nursing and case management services. The inpatient program is designed for patients with a variety of rehabilitation needs, offering general inpatient medical rehabilitation and three specialized programs: brain injury rehabilitation, stroke rehabilitation and spinal cord injury rehabilitation. In 2020, there were 13 rapid response events, and in 2021 there were 21. In 2022, there were 29. Typically, there were five to seven staff members consisting of nurses, therapy staff and two to three physicians at these rapid response events who use one of the three crash carts distributed at various locations throughout the hospital.

### Pre- & post-intervention questionnaire

A pre-intervention (PreIQ) and post-intervention questionnaire (PostIQ) (Supplementary 1: pre- and post-intervention questionnaire) was designed encompassing assessment of the ability to hear the rapid response announcement, comprehension of roles during a rapid response, and awareness of available equipment. The questionnaires, administered in English and employing a Likert scale, were distributed in-person to ARH clinical staff [18]. The targeted participants comprised of clinical staff actively engaged in the hospital's RRS, and the aim was to gauge their perspectives on the RRS at the hospital. Staff included physicians, physician assistants, charge nurses and residents. The questionnaire was not tested before its use; however, all authors reviewed and agreed that the questionnaire was adequate. The questionnaire was based on previously published instruments that gauged similar outcome measures [13]. To assess the impact of the interventions, the same questionnaires were distributed post-intervention. PreIQs were distributed in February 2021 and PostIQs December 2022, with a 100% response rate for both PreIQ and PostIQ. Due to the anonymous nature of the questionnaires, we cannot guarantee that the respondents before and after the intervention are consistent or identical.

### Intervention

The intervention process consisted of changes that were implemented to carry out the study. Crash carts were evaluated before intervention. Lack of standardization among crash carts were seen and intervention involved stocking crash carts with the Nursing Manager (Supplementary 2: crash cart inventory checklist). To address the problems with announcements, new speakers were installed in clinical staff workrooms and pagers were distributed to all clinical staff. Orientation to these changes was conducted in-person and virtually, and in-person drills were performed to ensure that all ARH clinical staff knew code protocols as well as their own role assignments during a rapid response event (Supplementary 3: bedside code roles).

### Statistics & analysis

Descriptive statistics were employed to provide a comprehensive overview of response to improve announcements, crash cart standardization and role assignments. Statistical analysis was performed using GraphPad Prism Version 9.3.1 (350) for macOS, GraphPad Software, MA, USA, www.graphpad.com.

## Results

### Questionnaire characteristics

A total of 15 PreIQs were completed by ARH clinical staff before implementation of this study. 15 PostIQs were completed by hospital clinical staff after the implementation of the interventions, making a total of 30 questionnaires completed between pre- and post-intervention.

### PreIQ results

**Figure 1.**
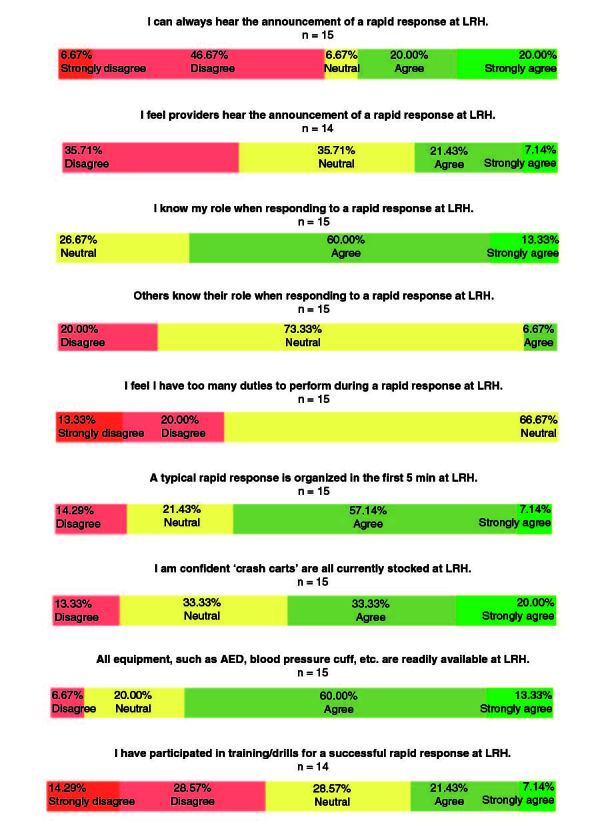
Pre-Intervention Questionnaire (PreIQ) results. Questions pertained to announcements of the rapid response, role assignments, crash cart stocks and training drills. 15 ARH clinical staff completed the PreIQ. AED: Automated external defibrillator; LRH: Lovelace UNM Rehabilitation Hospital.

Figure 1 illustrates the results of the PreIQ. Over 53% of surveyed ARH clinical staff disagreed or strongly disagreed that they could always hear the announcement of a rapid response. Approximately 36% reported concerns about other clinical staffs' ability to hear the announcement of a rapid response. Most clinical staff (73%) agreed or strongly agreed that they knew their roles, but 20% disagreed, while another 73% were neutral, when asked if others knew their role. Approximately 33% disagreed or strongly disagreed that they had too many duties to perform during a rapid response. Over 64% agreed or strongly agreed that rapid responses were organized within the first 5 minutes at the ARH. 53% of clinical staff stated that they are confident that “crash carts” were all stocked while 73% of clinical staff agreed or strongly agreed that equipment such as automated external defibrillator (AED) and blood pressure cuffs were readily available in the hospital. Only 29% reported participation in training or drills for a successful rapid response.

### PostIQ results

**Figure 2.**
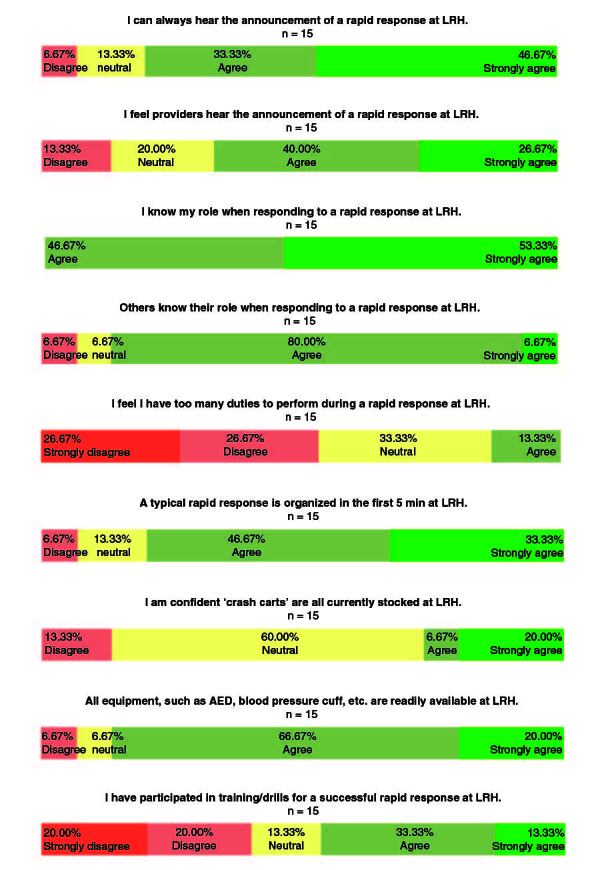
Post-Intervention Questionnaire (PostIQ) results. The same questions as the pre-intervention questionnaire (PreIQ) pertaining to announcements of the rapid response, role assignments, crash cart stocks and training drills were used for the post-intervention questionnaire (PostIQ). 15 ARH clinical staff completed the PostIQ. AED: Automated external defibrillator; LRH: Lovelace UNM Rehabilitation Hospital.

Figure 2 illustrates the results of the PostIQ. After intervention, substantially fewer respondents (only 7%) disagreed or strongly disagreed that they could always hear the announcement of a rapid response. Similarly, only 13% reported concerns about the ability of clinical staff to hear the announcement of a rapid response. All clinical staff indicated that they knew what their roles were during a rapid response, and only 7% disagreed and 7% were neutral when asked if others knew their role. Approximately 54% disagreed or strongly disagreed with the proposition that they had too many duties to perform during a rapid response. About 80% agreed or strongly agreed that rapid responses were organized within the first 5 min. Interestingly, only 27% of clinical staff expressed confidence that ‘crash carts’ were all stocked, reflecting a decrement from pre-intervention results. Nevertheless, 87% of clinical staff agreed or strongly agreed that equipment such as AED and blood pressure cuffs were readily available. Approximately 46% reported participation in training or drills for a successful rapid response.

## Discussion

Existing literature has showed that a successful rapid response involves leadership and mentorship as well as promotes continuous QI to ensure that clinical competency is met [^15-17^]. The goal of this QI study was to illustrate some barriers of the RRS that were present at our ARH and how interventions to address these barriers can positively affect the RRS. To our knowledge, there have been no papers that have analyzed the RRS at rehabilitation hospitals.

One of the barriers that was highlighted by the PreIQ was the inability to hear the announcement of a rapid response at the ARH. Over 50% of hospital clinical staff who completed the PreIQ reported that they were unable to hear the rapid response announcement. Not being able to hear announcements can lead to delayed rapid response activations, ultimately leading to outcomes such as increased hospital stay and mortality [19]. After installing speakers as well as distributing pagers to clinical staff, there was an increase in the amount of clinical staff who were able to hear the rapid response announcements.

As seen by previous studies, having a strong efferent limb consisting of an experienced multidisciplinary team is essential to the success of an RRS [8]. We addressed this in our study by implementing rapid response training and drills with ARH clinical staff. As seen in the PostIQ results, our interventions led to improvements with knowledge of role assignments, duty distribution and recognition of a quick rapid response activation. It is interesting that prior to the intervention, many ARH clinical staff knew their roles during a rapid response but felt neutral or disagreed that other individuals knew their roles. With the intervention, most clinical staff recognized what roles their colleagues had during a rapid response. More clinical staff also reported that they did not feel like they had too many duties during a rapid response, indicating that the intervention may have helped with highlighting what duties were designated for specific roles. There was also a small increase in individuals from the PostIQ reporting that organization of rapid responses occurred within 5 min at our hospital compared with the PreIQ. This may be due to a combination of both the implementation of the new announcement systems as well as the training and drills that were conducted for ARH clinical staff. As indicated by the PostIQ, the training and drill interventions were able to impact more ARH clinical staff compared with before the interventions as shown by the PreIQ (28% vs 46%).

Of note, a smaller proportion of clinical staff felt confident that the ‘crash carts’ were appropriately stocked post-intervention. It is possible that this reflects an increased perspicacity for the RRS including crash cart preparedness. More short-duration distributed training may be effective in increasing rapid response knowledge and training participation among hospital clinical staff [20].

This study has several limitations. One limitation is that this was done at a single ARH. We recognize the potential of this study having selection and indication bias in terms of individuals selected for questionnaires. Another limitation is the small sample size for the questionnaire results. Our data was also collected using a two-point, before-and-after method and thus presents a limited duration of follow-up after the intervention.

## Conclusion

Continuous QI may be an effective means of improving the RRS in hospitals including rehabilitation hospitals. Future directions to include continuous intervention and long-term follow-up will also be required to demonstrate a more sustained pattern of change. Future studies with a larger sample size will be needed to test generalizability. Incorporating increased frequency of rapid response training and drills for hospital clinical staff will also be useful in analyzing the effects of standardizing training on the quality of rapid responses.

## Supplementary Material

Supplementary files S1-S3

## Notes

To view the supplementary data that accompany this paper please visit the journal website at: www.future-science.com/doi/suppl/10.2144/fsoa-2023-0162
