# Supplementary figures and images for: Improvement of the rapid response system at an acute rehabilitation hospital in New Mexico
Source: Future Sci OA. 2024 May 24;10(1):FSO950. doi: 10.2144/fsoa-2023-0162 (PMC11152583; doi:10.2144/fsoa-2023-0162)

Supplemental 1:


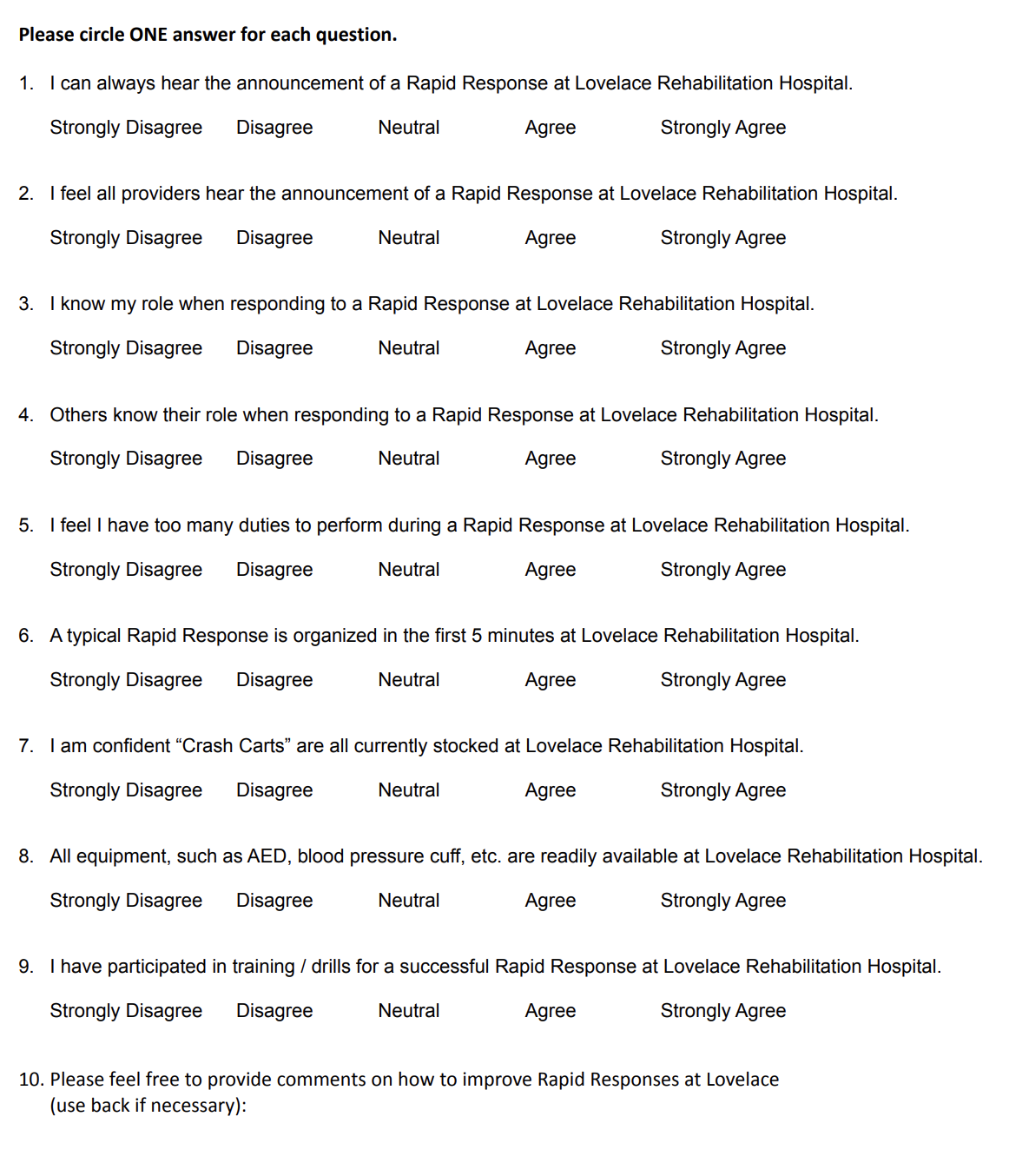

Supplement: Supplementary files S1-S3 [file IFSO_A_2342079_SM0001.zip › Code_Blue_REACTS_Manuscript_-_Supplemental_1.docx]

Supplemental 3:


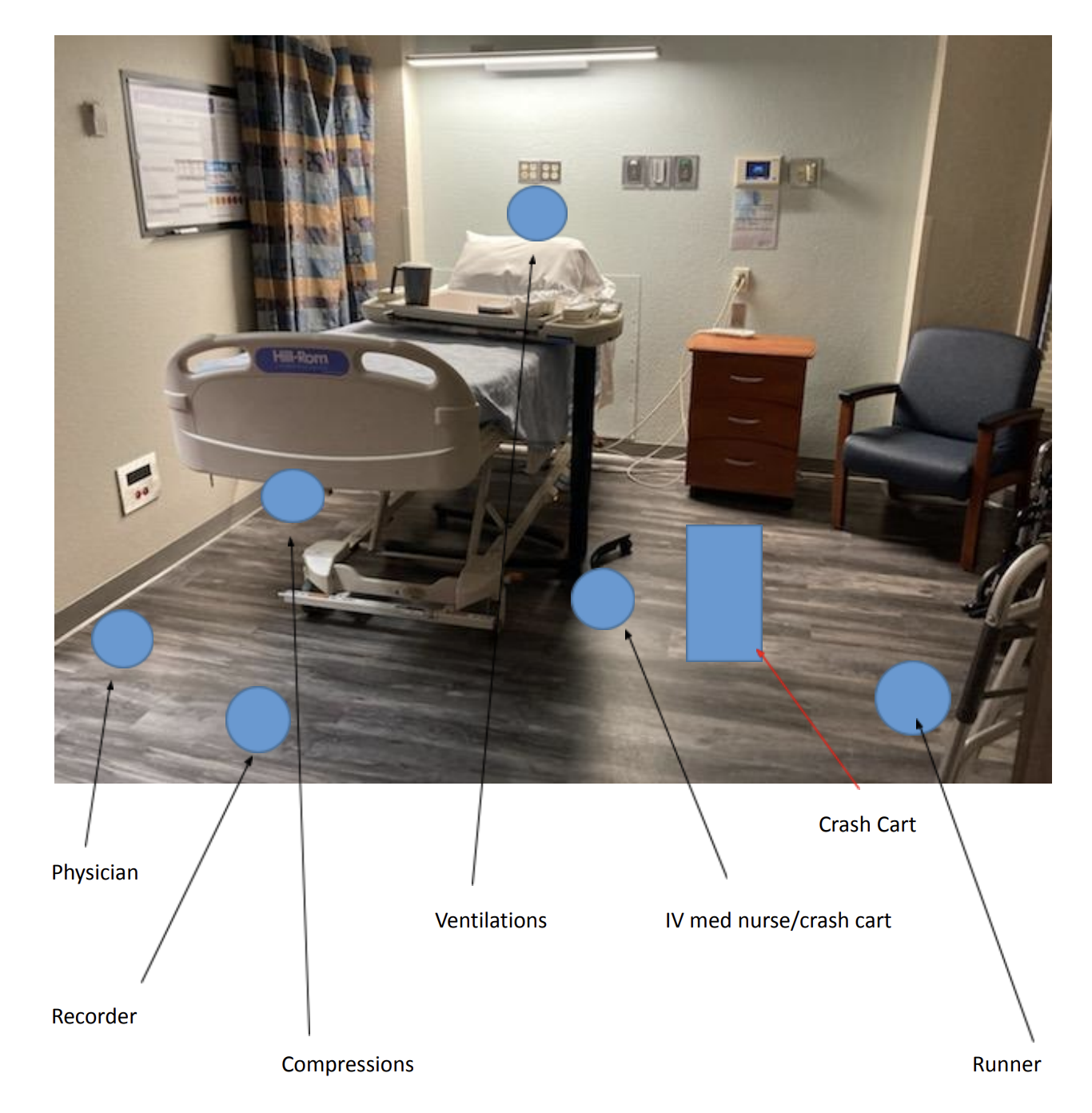

Supplement: Supplementary files S1-S3 [file IFSO_A_2342079_SM0001.zip › Code_Blue_REACTS_Manuscript_-_Supplemental_3.docx]
